# Evidence of liquid–liquid transition in glass-forming La_50_Al_35_Ni_15_ melt above liquidus temperature

**DOI:** 10.1038/ncomms8696

**Published:** 2015-07-13

**Authors:** Wei Xu, Magdalena T. Sandor, Yao Yu, Hai-Bo Ke, Hua-Ping Zhang, Mao-Zhi Li, Wei-Hua Wang, Lin Liu, Yue Wu

**Affiliations:** 1 School of Materials Science and Engineering, and State Key Laboratory of Material Processing and Die and Mould Technology, Huazhong University of Science and Technology, 430074 Wuhan, China; 2 Wuhan National High Magnetic Field Center, Huazhong University of Science and Technology, 430074 Wuhan, China; 3 Department of Physics and Astronomy, University of North Carolina, Chapel Hill, North Carolina 27599-3255, USA; 4 Institute of Physics, Chinese Academy of Sciences, Beijing 100190, China; 5 Department of Physics, Renmin University of China, Beijing 100872, China

## Abstract

Liquid–liquid transition, a phase transition of one liquid phase to another with the same composition, provides a key opportunity for investigating the relationship between liquid structures and dynamics. Here we report experimental evidences of a liquid–liquid transition in glass-forming La_50_Al_35_Ni_15_ melt above its liquidus temperature by ^27^Al nuclear magnetic resonance including the temperature dependence of cage volume fluctuations and atomic diffusion. The observed dependence of the incubation time on the degree of undercooling is consistent with a first-order phase transition. Simulation results indicate that such transition is accompanied by the change of bond-orientational order without noticeable change in density. The temperature dependence of atomic diffusion revealed by simulations is also in agreement with experiments. These observations indicate the need of two-order parameters in describing phase transitions of liquids.

## Abstract (web summary)

Non-density driven liquid-liquid transition has been predicted in theories, but direct experimental verification is challenging because liquid often remains metastable at transition temperature. Here, Xu *et al.* provide evidence in a lanthanum-based metallic glass above its liquidus temperature.

## Graphical abstract

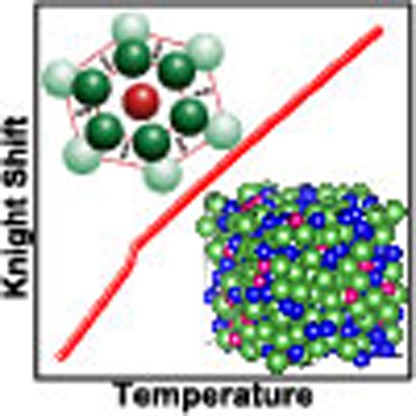

A liquid of certain complexity could assume different liquid phases of the same composition, a phenomenon called the liquid–liquid transition (LLT)^1^,^2^,^3^. So far, reports of LLT in experiments are quite rare and mostly limited to systems with unique electronic structures. For instance, cerium atom is known to assume either a trivalent 4*f*^1^(5*d*6*s*)^3^ or a tetravalent 4*f*^0^(5*d*6*s*)^4^ electronic structure, with the latter having a much smaller ionic size favoured under high pressure^4^. Polymorphism of pure Ce metal^4^,^5^ is well known and a LLT with a 14% change in density was observed in liquid Ce metal at 13 GPa^6^. Theoretical studies of LLT using two-scale spherically symmetrical ramp potential^7^ captures the basic features of such LLT where substantial structural changes of first nearest neighbours take place. The phase transition of liquid to supercritical fluid observed in pure phosphorus above 1 GPa is also related to drastic changes of first nearest-neighbour structures and electronic configurations of P atoms, accompanied by an estimated 40% change in density^8^,^9^,^10^. An important question is whether density is always the dominant order parameter associated with LLT. Could LLT be a generic phenomenon that can also take place in liquids containing no elements such as Ce with unique electronic structures? The two-order-parameter description of liquids points out that thermodynamic properties of liquids cannot be described sufficiently by density *ρ* alone and additional order parameters such as the local bond order parameter *S* are required^11^,^12^,^13^. It was suggested that LLT associated with the order parameter *S* is a generic property of liquids although its observation in practice can be difficult when the LLT temperature *T*_LL_ situates deep in the supercooled liquid region or at negative pressure^11^,^12^.

Recently, a LLT was reported in glass-forming liquid of Zr_41.2_Ti_13.8_Cu_12.5_Ni_10_Be_22.5_ (Vit1) where no substantial change of volume related to LLT was detected^14^. However, since this LLT is in the undercooled regime below the liquidus temperature *T*_liq_, it is difficult to obtain unambiguous signatures of first-order phase transition such as the temperature dependence of the incubation time associated with the phase transition. To unambiguously demonstrate the presence of LLT and rule out the potential influence of crystallization, it is desirable to observe LLT above *T*_liq_. Furthermore, to reveal the nature of the LLT in such systems, it is imperative to establish positive confirmation of the LLT by *ab initio* molecular dynamic (MD) simulations^15^,^16^, which allow detailed analysis of structural changes associated with the LLT.

In this work, we observe such LLT with transition temperature *T*_LL_ above *T*_liq_ in metallic glass-forming liquid La_50_Al_35_Ni_15_ that contains no elements such as Ce or P; the detection is made by high-temperature ^27^Al NMR, which is very sensitive to the changes of atomic structures and dynamics of liquids^17^,^18^. The liquid on LLT exhibits a sudden change in the temperature dependence of dynamics as reflected by a sharp change in the temperature dependence of NMR observables. In addition, *ab initio* MD simulations also reveal an abnormal temperature-dependent behaviour of atomic diffusion in the temperature range of LLT, in good agreement with experimental observations. Such agreement between experiments and *ab initio* MD simulations provides an opportunity to elucidate the nature of atomic structural changes associated with the LLT. It is found that while the change in density is insignificant, the bond-orientational order (BOO) parameter represented by 
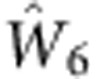
 (see Methods) exhibits a substantial drop with decreasing temperature in the temperature range of the experimentally observed *T*_LL_. This is consistent with the LLT theory based on the two-order-parameter description of liquids^11^,^12^. Such changes of local structures and dynamics with temperature revealed by *ab initio* MD simulations are also shown to be consistent with changes observed by NMR based on the view of the potential energy landscape (PEL)^19^,^20^,^21^.

## Results

### Phase transition above the liquidus temperature

**Figure 1:**
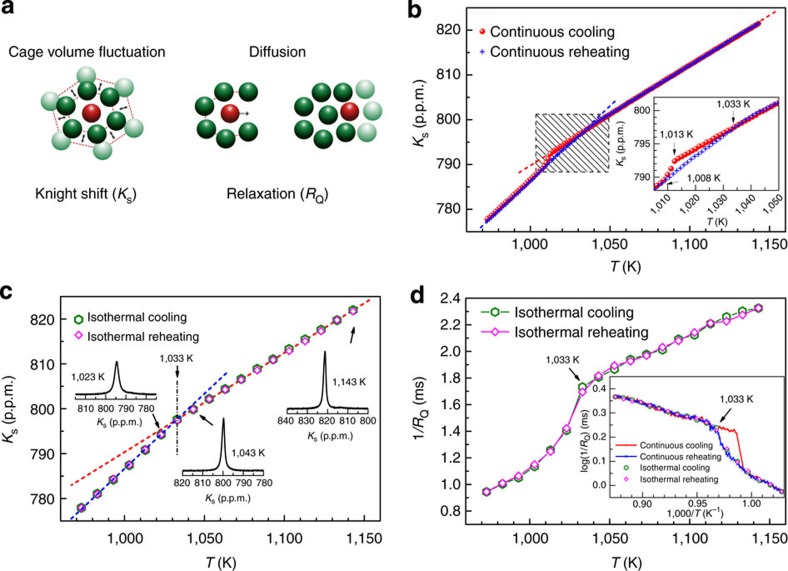
Changes of structures and dynamics in the melt. (**a**) Illustration of NMR technique. Cage volume fluctuation and atomic diffusion from one cage to another in metallic liquids can be detected by the temperature dependence of Knight shift (*K*_s_) and quadrupolar spin–lattice relaxation rate (*R*_Q_), respectively. (**b**) *T* dependence of ^27^Al *K*_s_ during continuous cooling and reheating in the temperature interval of 973–1,143 K. The red dashed line represents the fitting curves of *K*_s_ versus *T* with a slope of 0.22 p.p.m. K^−1^ and the blue dashed line represents the fitting curve with a slope of 0.33 p.p.m. K^−1^. The two fitting curves intersect at 1,033 K. The shaded region with hysteresis is magnified in the inset with three characteristic temperatures indicated by black arrows. (**c**) A series of isothermal measurements with consecutively decreasing temperatures down to 973 K followed by a series of isothermal measurements under consecutively increasing temperatures up to 1,143 K. The two fitting curves (red and blue dotted lines) with the slope of 0.22 and 0.33 p.p.m. K^−1^ are plotted together with the intersection at 1,033 K. Three typical ^27^Al NMR spectra taken at different temperatures are also shown. (**d**) *T* dependence of quadrupolar relaxation time 1/*R*_Q_ measured under isothermal cooling and isothermal reheating in the temperature interval of 973–1,143 K. Qualitative change in the temperature dependence of 1/*R*_Q_ occurs at 1,033 K. The inset shows the Arrhenius plots of log(1/*R*_Q_) versus 1,000/*T* measured under isothermal and continuous cooling/reheating cycles. The plots of log(1/*R*_Q_) versus 1,000/*T* measured under continuous cooling/reheating cycle shows the hysteresis occurring in the temperature range of 1,008–1,033 K due to the expected undercooling effect.

La_50_Al_35_Ni_15_ glassy rod was used as the sample in this work (see Methods for sample preparation). High-temperature differential scanning calorimetry (at a heating rate of 10 K min^−1^) revealed a glass transition temperature (*T*_g_) of 528 K and liquidus temperature (*T*_liq_) of 970 K for the glassy system (see Supplementary Fig. 1). *In situ* high-temperature ^27^Al NMR experiments were carried out above *T*_liq_ in a magnetic field of 9.4 T using a home-made high-temperature NMR probe^22^,^23^. The melted sample cut from the glassy rod is a 10 mg single droplet, ensuring temperature homogeneity and sensitive detection of phase transition events. The ^27^Al NMR spectrum at each temperature above *T*_liq_ is a single narrow peak of Lorentzian shape. As discussed later, the peak position of spectrum representing the Knight shift (*K*_s_) is sensitive to changes of structures and amplitude of cage volume fluctuations^17^, while the peak width is sensitive to diffusion^18^ as illustrated in Fig. 1a and b plots the *K*_s_ versus temperature *T*. Here the glassy sample (see Methods) was first heated to 1,143 K, held there for 2 h, and then cooled down continuously to 973 K at a constant cooling rate of 1 K min^−1^. NMR spectra were taken every 1 K interval during such cooling process where each NMR spectrum was acquired in <20 s (see Methods). After cooling down to 973 K the sample was held there for 2 h and was then reheated to 1,143 K at a constant heating rate of 1 K min^−1^. NMR spectra were taken again at every 1 K interval during the reheating process and the corresponding *K*_s_ versus *T* was also shown in Fig. 1b. The cooling curve of *K*_s_ varies linearly with *T* from 1,143 K down to 1,013 K with a slope of 0.22 p.p.m. K^-1^, followed by a sharp drop at 1,013 K within a narrow temperature range of 5 K (see the inset in Fig. 1b), and then continues with a linear *T*-dependence down to 973 K with a slope of 0.33 p.p.m. K^−1^. The continuous reheating curve of *K*_s_ reproduces the values of the cooling curve except in the temperature range of 1,008–1,033 K where hysteresis occurs. The error of *K*_s_ measurement at a given temperature is smaller than 0.2 p.p.m. (see Supplementary Fig. 2). The effect of hysteresis is a few p.p.m., much larger than experimental errors. Therefore, the hysteresis is consistent with the undercooling of a first-order phase transition. Above results were further evaluated by isothermal measurements. After completing the continuous reheating experiment described above, the sample was kept at 1,143 K for 4 h. NMR spectra were then taken at consecutively decreasing temperatures; at each temperature the system was equilibrated for 4 h before taking the NMR spectrum. Such isothermally measured *K*_s_ on cooling is plotted versus *T* in Fig. 1c along with three typical NMR spectra at 1,143, 1,043 and 1,023 K as examples. Subsequently, after reaching the lowest temperature of 973 K, the sample was reheated again and NMR spectra were taken at consecutively increasing temperatures; at each temperature the system was equilibrated for 4 h before taking the NMR spectrum. Such isothermally measured *K*_s_ on reheating is also plotted versus *T* in Fig. 1c. Similar to the continuous measurements, the isothermal cooling and reheating curves match perfectly with each other. A kink with slope change from 0.22 p.p.m. K^−1^ in high-temperature regime to 0.33 p.p.m. K^−1^ in low-temperature regime can be clearly identified at 1,033 K. This temperature can be approximately taken as the transition temperature for the observed phase transition. It is of note that there is no change of NMR peak intensity (the integrated area of the peak) above and below 1,033 K. This suggests that all Al atoms belong to a single macroscopic phase whether it is above or below the phase transition temperature at 1,033 K. It is also noted that the temperature dependence of isothermal *K*_s_ is completely reversible during cooling and reheating after more than 10 cycles, implying that the changes in stoichiometric composition is negligible during the whole testing process; furthermore, the alloy ingot preserves a metallic shiny surface after 10 cycles of cooling/reheating ruling out oxidation effects.

### Phase transition kinetics

**Figure 2:**
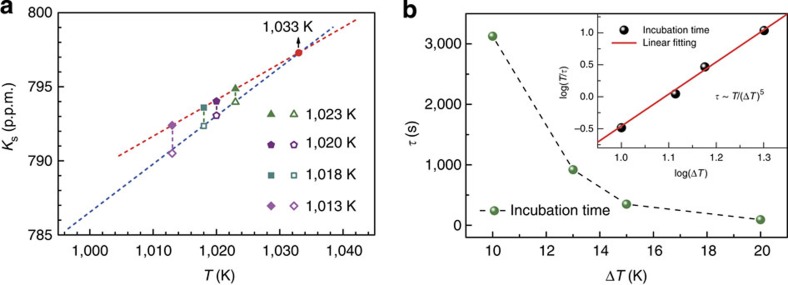
Kinetics of the LLT. (**a**) *K*_s_ at four chosen undercooled temperatures fast cooled from the same initial temperature of 1,143 K. The solid and open symbols represent the data before and after the transition, respectively. (**b**) Average (of three sets of data) incubation time *τ* versus degree of undercooling Δ*T*=1,033 K−*T*. The inset plots log(*T/τ*) versus log(Δ*T*) and the slope of the straight line is 5.

To further elucidate the nature of the phase transition, we performed the isothermal NMR experiments to study the temperature dependence of the incubation time associated with the phase transition. After the sample was held at 1,143 K for 2 h, it was cooled down rapidly (∼20 K min^−1^) to a chosen undercooled temperature below 1,033 K (herein, 1,033 K is estimated the transition temperature without undercooling on the basis of the results in Fig. 1b,c). At each undercooled temperature, the incubation time *τ* of the phase transition can be measured by monitoring isothermally the occurrence of phase transition by taking NMR spectrum every 4 s. As shown in Fig. 2a, the *K*_s_ values measured at 1,023, 1,020, 1,018 and 1,013 K before the transition are on the straight line extended from the values of *K*_s_ versus *T* above 1,033 K measured during isothermal cooling and reheating process, indicating the existence of undercooling of the high-temperature liquid (taking into account that 1,033 K is the transition temperature); after certain incubation time *τ* the transition takes place and *K*_s_ jumps to the values as expected from the isothermal cooling and reheating measurement below 1,033 K (see the blue line). The phase transition incubation time at four undercooled temperatures was measured systematically. Here incubation time measurements were repeated three times and the average of the three measured *τ* values for each temperature is plotted versus undercooled degree (Δ*T*=1,033 K−*T*) in Fig. 2b. The timescale of the undercooling effect with Δ*T*>20 K is too short to be captured by the current experimental approach. The incubation time drops quickly from 3,128 to 93 s by increasing Δ*T* from 10 to 20 K, consistent with a first-order phase transition. The incubation time of phase transitions in the liquid state can be very long under small degree of undercooling in bulk metallic glass (BMG)-forming systems^24^,^25^. The change can be described empirically by *τ*∝*T*/(Δ*T*)^*m*^ with *m*=5 as shown in the inset of Fig. 2b. Homogeneous nucleation process of crystallization was also found to follow a power-law dependence^26^. The issue of incubation time is very complex. It is still under intensive investigation even for liquid–solid transition in very simple systems such as CuZr alloy^27^. Since the present case is a liquid–liquid transition rather than liquid to solid transition, the nucleation and growth processes can be quite different from traditional crystallization phenomena and more quantitative understandings of incubation time require more experimental and theoretical studies of LLT in the near future.

### Changes of atomic diffusion

To further characterize the observed phase transition, it is important to investigate the change of dynamics associated with the phase transition. Atomic diffusion is an important characteristic of metallic liquid reflecting its structure and dynamics^28^. For quadrupolar nuclei such as ^27^Al with spin quantum number *I*=5/2, atomic diffusion gives rise to efficient spin–lattice relaxation due to diffusion-induced fluctuations of the electric-field gradient (EFG)^18^,^29^,^30^. In the fast motion limit, the quadrupolar spin–lattice relaxation rate *R*_Q_ can be expressed^31^ as 
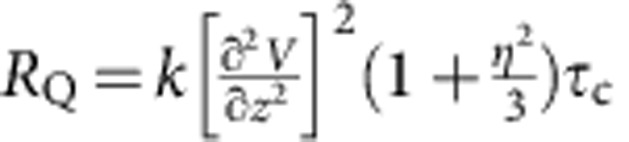
 where *k* is a constant, *η* is the asymmetry parameter, 
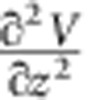
 is the maximum of the EFG tensor at Al site and *τ*_c_ is the correlation time of the EFG at Al sites, which measures the persistence time of the atomic cage surrounding the Al atom^18^,^32^,^33^. It is of note that diffusion of all atoms neighbouring Al could cause fluctuations of the EFG tensor at Al sites. Smaller diffusion coefficient *D* leads to less frequent fluctuations of the EFG tensor and thus longer *τ*_c_. Here *D* is proportional to 1/*R*_Q_ in the melt as being already established previously in other metallic liquids^34^,^35^. *R*_Q_ can be derived from the measured spectral linewidth (see Methods). Figure 1d shows the data of 1/*R*_Q_ obtained from isothermal cooling and reheating, indicating that 1/*R*_Q_ decreases with decreasing temperature in the region from 1,143 to 973 K, but a qualitative change occurs at 1,033 K. Here 1/*R*_Q_ decreases nearly linearly with decreasing *T* above 1,033 K and shows a much steeper decrease with decreasing *T* after 1,033 K. The sharp change in the temperature dependence of 1/*R*_Q_ at 1,033 K indicates that atomic diffusion coefficient *D* exhibits an increased activation energy below 1,033 K, as reflected by the slope change at 1,033 K in the Arrhenius plots of log(1/*R*_Q_) versus 1,000/*T* (inset of Fig. 1d). Besides the isothermal data of 1/*R*_Q_, the inset of Fig. 1d also shows log(1/*R*_Q_) versus 1,000/*T* obtained in the continuous cooling/reheating measurements; it reveals the same trend as that of the isothermal measurement except the hysteresis occurring in the temperature range of 1,008–1,033 K due to the undercooling effect captured by the fast cooling rate. This hysteresis phenomenon is in perfect agreement with the temperature dependence of *K*_s_ measured in the continuous measurement shown in Fig. 1b.

### Structure and dynamics from *ab initio* MD simulations

**Figure 3:**
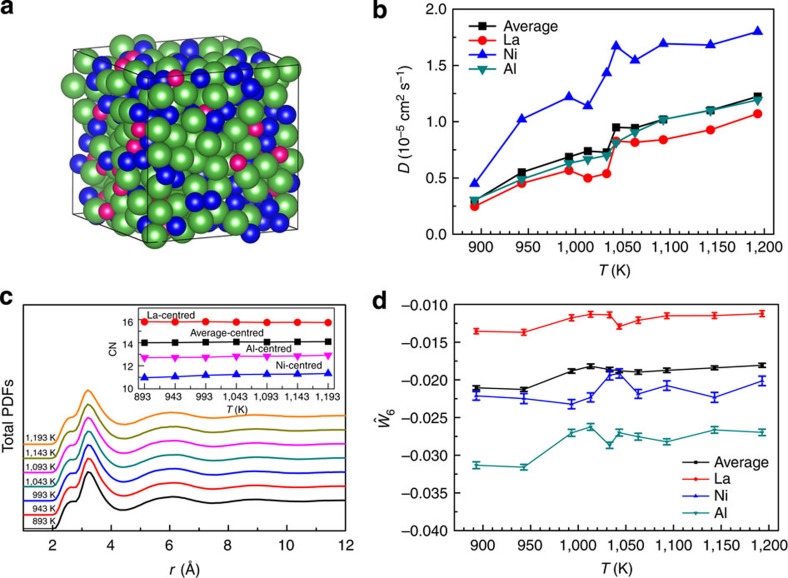
*Ab initio* MD simulations. (**a**) A typical atomic configuration equilibrated in the liquid state at 1,143 K modelled by *ab initio* MD simulations. Green, blue and red balls represent La, Al and Ni, respectively. (**b**) Temperature-dependent diffusion coefficient of La_50_Al_35_Ni_15_ liquid, which shows qualitative changes around 1,043 K. (**c**) Total pair distribution functions (PDFs) of La_50_Al_35_Ni_15_ liquid at different temperatures. The inset shows the variation of coordination number (CN) with temperature. (**d**) Temperature dependence of the average 
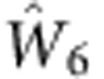
 of all three elements and 
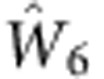
 of individual element. The error bars are shown along with the data symbols. The error bars were estimated from the s.d. of 
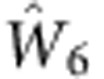
 in two independent MD simulations.

To reveal the structural origin for the observed phase transition above *T*_liq_, we pursued the atomic structure and dynamics of the liquid via *ab initio* MD simulations. In *ab initio* MD simulation, the atomic forces are determined with the first-principle calculations without using any experimental input or empirical potentials (see Methods). So far, plenty of studies have demonstrated that the independent *ab initio* MD simulations successfully obtain very reasonable and reliable atomic structures for liquid and amorphous materials^36^,^37^,^38^,^39^. *Ab initio* calculations are also capable of monitoring the evolutions of structure and dynamics in the liquid during the cooling process, so that direct comparison to experimental results of liquid dynamics can be made and a link between dynamics and atomic structures of liquids can be established. Figure 3a shows a typical simulated atomic configuration. To obtain dynamic information of liquids, we analysed the self-diffusion coefficient (*D*) of each element in the liquid at various temperatures based on the calculation of the mean-square displacement (MSD; see Methods). As shown in Fig. 3b, a clear slope change of the average *D* is observed around 1,040 K. Among the three elements in the alloy, Ni and La (especially Ni) exhibit more pronounced slope change, while Al displays only a slight change; this suggests that Ni makes a major contribution to the abnormal dynamics of the liquid around 1,040 K. Owing to the thermal fluctuations in high-temperature MD simulations, it is difficult to reveal small discontinuous change by *ab initio* MD. However, continuous change of *D* but discontinuous change of its temperature dependence are typical for LLT according to previous theoretical studies^40^,^41^. As discussed above on the change of 1/*R*_Q_ versus *T*, diffusion of all atoms neighbouring Al will cause fluctuations of the EFG tensor at Al sites such as that of Ni. Thus, the slower temperature dependence of the self-diffusion coefficient of Ni in the high-temperature region is fully consistent with the slower temperature dependence of 1/*R*_Q_ above *T*_LL_. Furthermore, the transition temperature in dynamics from the *ab initio* MD simulations is very close to the temperature of 1,033 K in the phase transition observed by NMR. Such high consistency between experiments and *ab initio* MD simulations further validates the LLT observed in the liquid. It also provides an opportunity to link the LLT with the atomic structural evolution.

To investigate the atomic structural evolution, the total pair distribution functions and the coordination number of each element at various temperatures were calculated and are shown in Fig. 3c. It can be seen that total pair distribution functions and coordination numbers do not show any noticeable change with temperature, indicating that the change of the density in the liquid within the investigated temperature range is very small. Therefore, it can be concluded that the observed LLT in the present La-based alloy system is not a density-driven phase transition and is very different from what was observed in Ce^6^ and P^8^. Some other order parameters related to local atomic structures have to be identified for understanding the mechanism of LLT revealed in above analysis^12^.

To find out the underlying local structure change, we did the analysis of the BOO based on spherical harmonics, which is a quantitative description of the different bond-orientational symmetry around a central atom^42^ and is a sensitive measure to the change of local structures (see Methods). Specifically, 
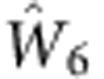
 is sensitive to the appearance of five-fold symmetry. 
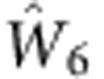
 values of face-centred cubic, hexagonal close-packed and icosahedron clusters are −0.01316, −0.01244 and −0.16975, respectively. Figure 3d shows the temperature dependence of the averaged 
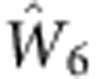
 and the individual contributions from La-, Ni-, Al-centred atomic clusters with error bars in La_50_Al_35_Ni_15_ melt. It is shown that below 1,050 K the system enters a temperature region where the individual 
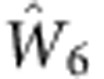
s change significantly until below 1,000 K. The values of the average 
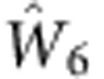
 change slightly between −0.018 and −0.019 in the temperature range above 1,050 K, but drops to −0.021 below 1,000 K, indicating that La_50_Al_35_Ni_15_ melt changes its state from one with lower fraction of five-fold symmetry above the transition region to one with higher fraction of five-fold symmetry below the transition region. The overall trend of increased five-fold symmetry from above to below the transition does not imply that the five-fold symmetry around all atoms have to increase at the same time. The preference of icosahedral order for one element could be at the cost of other elements because of correlations due to short- and possibly medium-range orders. The temperature-induced variations of 
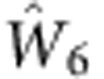
 of individual elements are coupled, and influence each other. In addition, Voronoi Tessellation analysis^43^ was performed to detect the changes in short-range order in La_50_Al_35_Ni_15_ melt (see Methods). The results also indicate that some major Voronoi clusters change subtly around the transition temperature region (see Supplementary Fig. 3).

## Discussion

**Figure 4:**
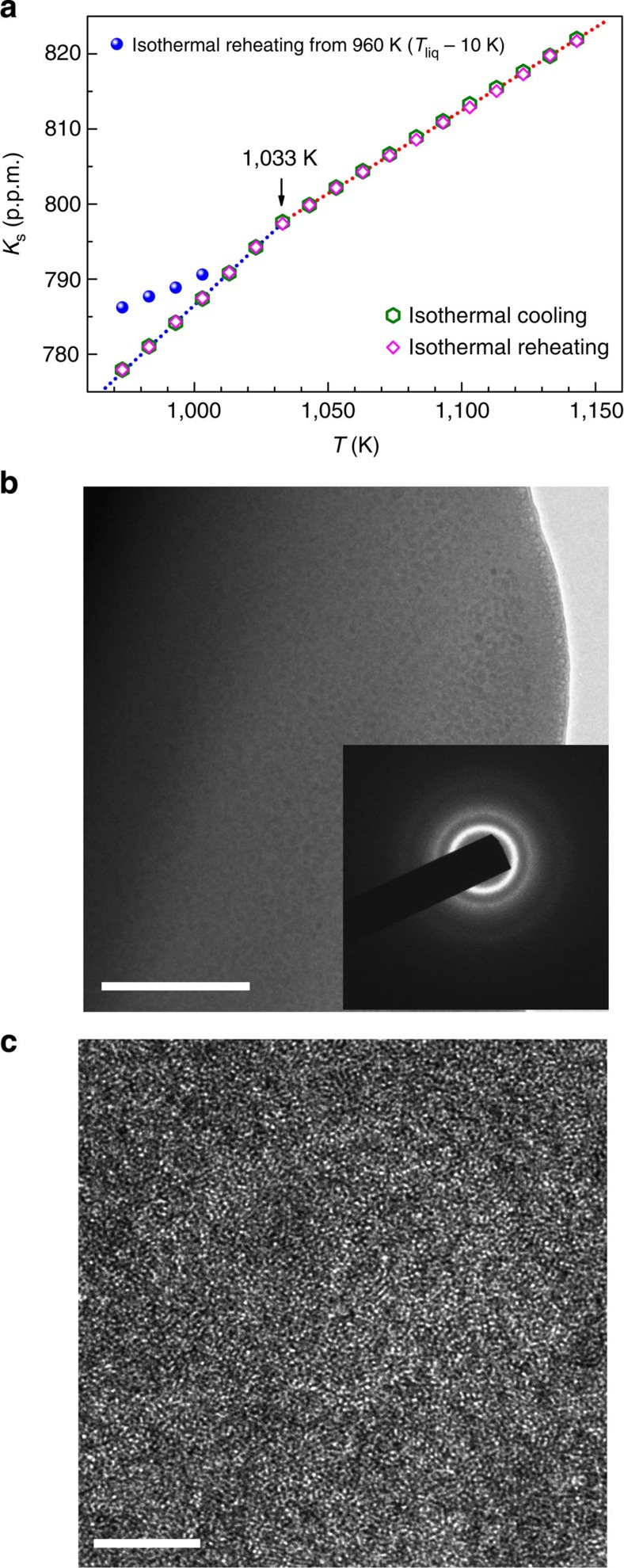
Evidences ruling out nanocrystallization and macroscopic phase separation. (**a**) The effect of crystallization at 960 K on *K*_s_ is shown with large deviations from the pure liquid data. (**b**) A larger scale TEM image of the glass ribbon obtained by melt-spinning method rapidly quenched from the melt at 1,003 K, which cooled from 1,143 K (see Methods). Scale bar, 100 nm. The inset shows the selected-area electron diffraction pattern. (**c**) High-resolution TEM image of the ribbon without obvious periodic contrast. Scale bar, 5 nm.

The significance of the current experiment is that the observed phase transition around 1,033 K is significantly above *T*_liq_=970 K. This allows detailed isothermal measurements to obtain the evidence of first-order phase transition, such as the temperature dependence of the incubation time associated with the phase transition, and to elucidate the changes of dynamics associated with the phase transition without the influence of potential crystallization. The effect of crystallization can be easily detected by NMR when the sample is cooled below *T*_liq_. Once the sample was cooled down to 960 K (<*T*_liq_), as shown in Fig. 4a, the *K*_s_ value shows deviations when it was reheated above *T*_liq_. The deviation of *K*_s_ near *T*_liq_ is large and still remains after equilibrating for 4 h near *T*_liq_. This effect associated with crystallization is common in metallic alloys^44^. Crystallization produces phase-separated ingot, and thorough mixing to form homogeneous liquid near *T*_liq_ might be difficult due to many factors such as viscosity and gravity. This effect can be even more pronounced in BMG-forming alloys due to enhanced viscosity. However, thorough mixing can be achieved quickly at elevated temperatures^44^ such as 1,100 K in our case. To rule out possible nanocrystallization and macroscopic phase separation associated with the phase transition, a glass ribbon sample was made by melt spinning at high quenching rate of 10^5^–10^6^ K s^−1^ (see Methods) from 1,003 K (after cooling down from 1,143 K), which is below the phase transition temperature 1,033 K. Such rapidly quenched glass could capture the structure in the melt at 1,003 K such as the potential presence of nanocrystallization and macroscopic phase separation if they would have occurred. Figure 4b,c show transmission electron micrographs (TEMs) and high-resolution TEM of the prepared glass ribbon. The results show that the quenched sample is completely amorphous without any trace of nanocrystallization and macroscopic phase separation. In addition, there is also no evidence of phase separation by NMR. A single sharp Lorentzian peak of ^27^Al NMR was observed both before and after the transition without the change of peak intensity. Furthermore, *K*_s_ can be traced reversibly on cooling and reheating crossing the transition temperature 1,033 K over a relatively short time. Thus, liquid–liquid phase separation on macroscopic scale is very unlikely. Ostwald ripening and coalescence would make reversible mixing over a short time very difficult.

In metallic systems, the dominant contribution to *K*_s_ comes from the direct Fermi contact shift due to *s*-conduction electrons. The orbital effect for Al is very small compared with the Fermi contact contribution except in cases of very narrow gap semiconductors^45^. Even in such a case, the temperature dependence of the shift with *T* is very weak^45^, unlike in the present case where the shift changes over 100 p.p.m. Therefore, the observed temperature dependence is dominated by the Fermi contact interaction, which is also confirmed in other metallic glass-forming liquid^17^. In such a case, *K*_s_ can be expressed as 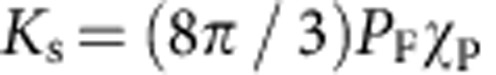 where *χ*_P_ is the Pauli paramagnetic susceptibility and *P*_F_ is the probability density of an electron wave function at the nucleus averaged over states at the Fermi level *E*_F_^46^,^47^. *χ*_P_ can be chosen as the volume susceptibility and electron wave function is then normalized over the unit volume. It is of note that direct numerical calculation of *K*_s_ requires knowing the precise value of the electron wave function at the nucleus and the calculation of the precise value of the Knight shift in metallic alloys by density-functional theory remains very challenging^48^,^49^. However, the importance in the current study is not the absolute value of *K*_s_ but its temperature dependence. The *K*_s_(*T*,*V*) is a function of the density (or the volume *V* with a constant number of atoms) and *T*. Since *V* is a function of *T* and pressure *P*, we have *K*_s_(*T*,*V*(*T*,*P*)) Therefore, the following thermodynamic relation holds^50^


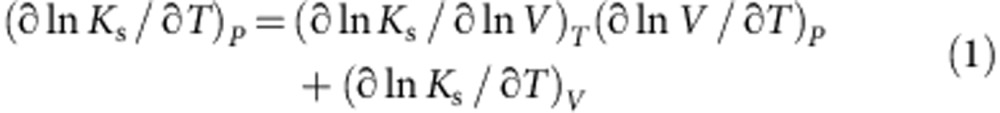


The first term on the right-hand side of equation (1) represents the change in *K*_s_ due to volumetric thermal expansion, and the second term represents the change in *K*_s_ due to temperature-induced dynamic variation. In this study, the measured value of (∂ln*K*_s_/∂*T*)_*P*_ is 2.8 × 10^−4^ and 4.1 × 10^−4^ K^−1^ above and below 1,033 K, respectively. (∂ln*K*_s_/∂*V*)_*T*_ is usually negative with a value of −1.0 for Al as determined from its pressure dependence^51^. The volumetric thermal expansion coefficient (∂ln*V*/∂*T*)_*P*_ is estimated to be ∼10^−4^ K^−1^ for La_50_Al_35_Ni_15_ liquid based on previously reported data^52^,^53^. Therefore, the first term in equation (1) would contribute about −10^−4^ K^−1^ to (∂ln*K*_s_/∂*T*)_*P*_ and the observed change of (∂ln*K*_s_/∂*T*)_*P*_ from 2.8 × 10^−4^ to 4.1 × 10^−4^ K^−1^ might come predominantly from the explicit temperature-dependent term (∂ln*K*_s_/∂*T*)_*V*_ in equation (1). The term (∂ln*K*_s_/∂*T*)_*V*_ is related to thermal fluctuations of atomic volume (or the fluctuations of nearest-neighbour atomic distance)^17^ and can be formally expressed as^50^


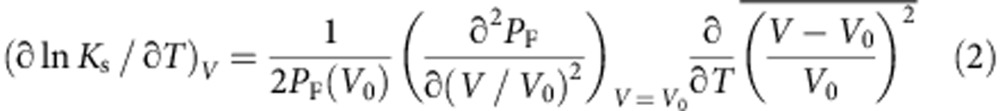


where *V*_0_ is the thermal equilibrium volume. The time-averaged mean squared fluctuation in volume can be expressed as 
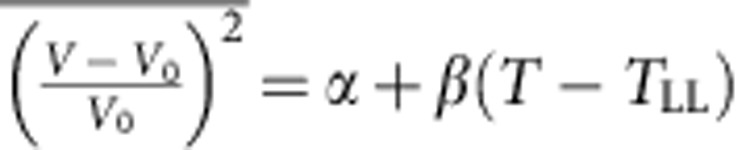
 using Taylor expansion, where *α* is the value of 
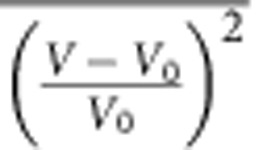
at *T*=*T*_LL_, and *β* is a coefficient reflecting the fluctuation amplitude of atomic volume in the system^17^,^50^,^54^, which is related to 
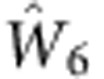
 and will be discussed later on. Similar vibration-induced linear temperature dependence was also observed previous in the chemical shift of high-temperature liquids^55^. Since the fluctuation amplitude of atomic volume always decreases with decreasing temperature in metallic liquids, that is, *β* is always positive, the observed *K*_s_ will decrease with decreasing *T* both in high-temperature and low-temperature liquids, irrespective of the presence of local five-fold symmetry represented by 
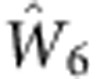
. In the liquid state, the relevant fluctuation to the NMR observation is Al atomic volume fluctuation before cage collapsing and is thus of high frequency^17^. 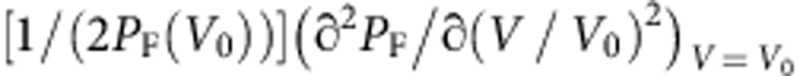 is on the order of 1 (ref. 50). Therefore, the observed change of (∂ ln *K*_s_/∂*T*)_*P*_ determined by the change of (∂ ln *K*_s_/∂*T*)_*V*_ is predominantly the result of the change of the coefficient *β*. The larger slope of *K*_s_ versus *T* below 1,033 K indicates that the magnitude of Al atomic volume fluctuations decreases faster with decreasing *T* below *T*_LL_ compared with that above *T*_LL_. Therefore, the steeper slope of *K*_s_ versus *T* below *T*_LL_ is predominantly related to a change of cage volume fluctuation around Al. In addition to *K*_s_, 1/*R*_Q_ is sensitive to the collapse of atomic cage and thus to diffusion^18^,^32^,^33^ (see Fig. 1a). Since diffusion of all atoms neighbouring Al could cause fluctuations of the EFG tensor at Al sites, the atomic diffusion *D* is proportional to 1/*R*_Q_. It is clear that the diffusion of atoms always decreases with decreasing temperature in metallic liquids, leading to a decrease of 1/*R*_Q_ with decreasing *T*. Since decreasing cage volume fluctuations would lead to lower success rate of cage escape and slower atomic diffusion, the steeper decrease of the amplitude of cage volume fluctuations versus *T* below *T*_LL_ (as measured by the steeper decrease of *K*_s_) would also lead to steeper decrease of diffusion (as measured by 1/*R*_Q_ shown in the inset of Fig. 1d). The temperature dependences of both *K*_s_ and 1/*R*_Q_ measure the dynamic properties with the former measuring cage volume fluctuation and the latter measuring the rate of cage collapsing (of atomic cages around Al atoms).

As discussed earlier, the change in density associated with the observed LLT is small, consistent with an earlier study of a metallic glass-forming alloy^14^. Therefore, the nature of LLT observed in La_50_Al_35_Ni_15_ melt is different from that observed in Ce (ref. 6) and P (ref. 8) where density exhibits drastic change. This indicates that density is not the dominant order parameter for describing the observed LLT in La_50_Al_35_Ni_15_ melt. The nature of LLT in La_50_Al_35_Ni_15_ melt is consistent with the two-order-parameter description of liquids proposed by Tanaka^11^,^12^. In this two-order-parameter theory, two order parameters are invoked in describing LLT, namely, density *ρ* and local bond order parameter *S* characterized by BOO. It is proposed that there exist distinct locally favoured structures that are energetically more favourable and its population is defined as the order parameter *S*. These locally favoured structures interact with the surrounding environment represented by a term *JS*(1−*S*) (*J* is the cooperativity strength) in the free energy, which could lead to cooperativity, and a thermodynamic phase transition in liquid could occur between two free energy valleys, which are determined by two distinct values of the *S* parameter. Therefore, a LLT could correspond to a sudden change of *S* associated with a change of BOO (see Supplementary Fig. 4)^11^,^12^. As demonstrated in the analysis of BOO versus *T* (see Fig. 3d), a significant decrease of the average 
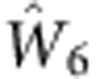
 indeed occurs before and after the LLT in La_50_Al_35_Ni_15_ melt, consistent with a substantial increase of locally favoured structures (that is, clusters with enhanced five-fold symmetry) below *T*_LL_.

In the PEL picture^19^,^20^,^21^, the liquid properties are associated with the manner in which a liquid system samples its inherent structures as a function of temperature. At high temperatures, the atoms with high kinetic energy are only weakly restricted and the liquid system is in the so-called free diffusion regime^20^. Here the liquid system samples a broad region of the PEL and the sampled average inherent structure energy is shown to depend only weakly on temperature^19^,^20^. Therefore, both cage volume fluctuations and diffusion rate decrease slowly with decreasing temperature as observed in simple metallic liquids^56^. However, as LLT occurs with decreasing temperature, the liquid possesses more locally energetically favoured structures with increased five-fold symmetry short-range order clusters (that is, more negative 
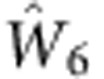
); atomic diffusions are now more restricted with more stable cage structures and the system enters into the PEL-influenced regime^18^,^19^,^20^. In such PEL-influenced regime, effects of energy barriers become important and the average energy of inherent structures is shown to decrease rapidly with decreasing temperature^19^,^20^. Similarly, as the inset of Fig. 1d shows, diffusion exhibits increased activation energies with decreasing *T* similar to previous NMR and diffusion studies of liquids^57^,^58^. Rapid decreases of cage volume fluctuations and diffusion rate are also expected in the regime where energy barriers play an important role as compared with the free diffusion regime. Therefore, steeper temperature dependences of *K*_s_ and 1/*R*_Q_ below *T*_LL_ are fully consistent with substantially increased locally favoured structures below *T*_LL_ as revealed by the change of 
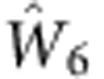
 in MD simulations.

In summary, the evidence of a LLT is identified by high-temperature ^27^Al NMR in glass-forming La_50_Al_35_Ni_15_ melt above its *T*_liq_. Since the LLT occurs above *T*_liq_, detailed experiments such as the dependence of the incubation time *τ* on the degree of undercooling Δ*T* can be carried out. The obtained experimental evidence is consistent with a first-order phase transition. In addition, NMR also reveals significant changes of atomic diffusion behaviour on LLT. Evidence supporting LLT was also obtained by *ab initio* MD simulations; changes of atomic diffusion behaviour was revealed over the same temperature region in agreement with NMR results. Such agreement between experiments and *ab initio* MD simulations provides a unique opportunity to reveal the structural origin associated with the LLT. *Ab initio* MD simulations show that the observed LLT is associated with changes in BOO and such changes do not produce significant change in density. The present result indicates the order parameter *S* closely related to the BOO characterized by the average 
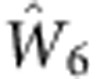
 could be the dominant order parameter associated with the observed LLT (instead of density) in this alloy system containing no special elements such as Ce and P. The observed LLT is consistent with the theory of LLT based on the two-order-parameter description of liquids.

## Methods

### Sample preparation

The La_50_Al_35_Ni_15_ BMG rods with diameter of 3 mm were obtained by water-cooled copper mould casting with high-purity ingredients of La, Al and Ni (purity>99.9 wt%) under argon atmosphere. Their amorphous nature was identified by X-ray diffraction and no crystallization peak was found. A single piece of such BMG sample weighted about 10 mg was cut from the as-cast BMG rod and used for the high-temperature NMR experiments. Experiments using different batches of samples give identical results. The samples were vacuum sealed in a quartz tube under high vacuum (better than 10^−5^ Pa) by a turbo pump after six times flushing with 99.9999% high-purity argon gas to prevent the sample from oxidation. The sample was supported on a magnesia-stabilized zirconia substrate that prevents the melt from touching the wall of quartz container. The sample remains in spherical shape and its surface remains shiny after more than 10 times cooling and heating cycles at high temperatures.

To obtain the glass ribbon of La_50_Al_35_Ni_15_ fast frozen from its liquid state below 1,033 K for TEM, a BMG sample was heated up to 1,143 K, held there for 2 min and then cooled down to 1,003 K. After holding the liquid at 1,003 K for 2 min, the melt was rapidly quenched by melt spinning with a copper wheel at a speed of 50 m s^−1^.

### High-temperature NMR experiments

The temperature was carefully calibrated and the gradient at the sample's position varied <0.3 K over the whole temperature range by pinpointing the melting point of pure copper and pure aluminum using their temperature dependence of *K*_s_. The accuracy of the temperature is ±2 K.

The high-temperature NMR experiments were carried out using a Bruker Avance III 400WB spectrometer with a magnetic field of 9.4 T. This gives a resonance frequency of 104.28 MHz for ^27^Al with spin quantum number *I*=5/2. A standard one pulse with the 90° pulse width of 10 μs was used for the experiments above *T*_liq_. Recycle delay of 20 ms was used for signal averaging. A total of 128 scans (about 20 s) were accumulated for each measurement during continuous cooling and reheating process at 1 K min^−1^. A total of 5,120 scans (about 640 s) were accumulated in the measurement at every temperature during isothermal experiments to get a better signal to noise ratio. For the measurement of incubation time with different degree of undercooling, a total of 32 scans (about 4 s) were accumulated to detect the start of the transition behaviour promptly. All ^27^Al NMR spectra were referenced to 1 mol l^−1^ Al(NO_3_)_3_ aqueous solution.

### Calculation of the quadrupolar relaxation rate

*R*_K,_ which is the contribution of the magnetic interaction associated with *K*_s_, was subtracted from the total spin–lattice relaxation rate (*R*) to obtain *R*_Q_. *R*_K_ can be estimated from the Korringa relation^47^


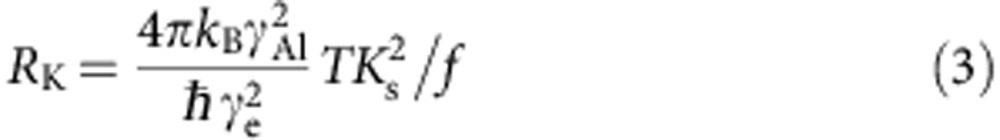


where *γ*_A1_and *γ*_e_ are the gyromagnetic ratios of the aluminum nucleus and the electron spin, respectively; *f* is the enhancement factor due to electron–electron interactions. In this study, *f* was estimated to be 2.3 according to the equation (3) based on the data of *K*_s_ at 323, 373, 423 and 473 K. In the fast motion limit, spin–lattice relaxation rate and spin–spin relaxation rate are the same, which can be extracted from the full width at half maximum (Δ*v*_1/2_) of the Lorentzian-shaped spectrum by Δ*v*_1/2_=*π*^−1^*R* (refs 18, 47).

### *Ab initio* MD simulations

The *ab initio* MD simulations were performed to achieve the atomic configurations of the liquid, using the projector augmented-wave method within the density-functional theory as implemented in the Vienna *ab initio* simulation package^59^. Canonical NVT (constant number, volume, temperature) ensembles containing 500 atoms in a cubic box with periodic boundary conditions were well equilibrated in the liquid state at 1,193 K for 2,000 time steps (each time step is 3 fs). The temperature was controlled with a Nose–Hoover thermostat^60^. Then the systems were cooled down to 893 K at a constant cooling rate of 0.05 K per step. In our simulations, the density of the system is determined as follows. At each temperature, the conjugated gradient method was used to obtain the inherent structure^61^ of the liquid. The density was adjusted to have a zero pressure of the inherent structure of the liquid. The inherent structure of the liquid at each temperature was further relaxed isothermally for 5,000 time steps for the analysis of the structure and dynamical properties. The *ab initio* MD simulations were carried out only at the *Г* point. Projected augmented plane waves^62^,^63^ with the generalized gradient approximation of Perdew and Wang (PW91) exchange-correlation potentials^64^ have been adopted.

### Calculation of self-diffusion coefficient *D*

The self-diffusion coefficient *D* in liquids was obtained by fitting the long time MSD at different temperatures after 15 ps isothermally annealing. MSD is defined as 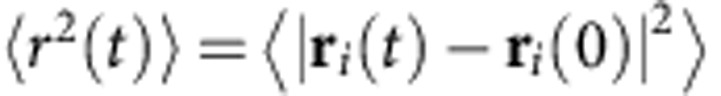. Here **r**_*i*_(*t*) is the atomic position of atom *i* at time *t* and 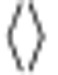 denotes ensemble average. The diffusion coefficient *D* can be calculated by 
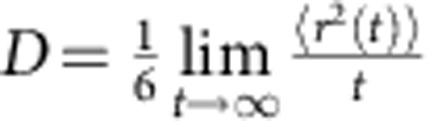
.

### Bond-orientational order parameter

To quantitatively describe the bond-orientational symmetry around centre atom, Steinhardt *et al.* developed BOO by employing spherical harmonics^42^. The BOO of atom *i* can be represented by the *l*-fold symmetry as a 2*l*+1 vector defined as


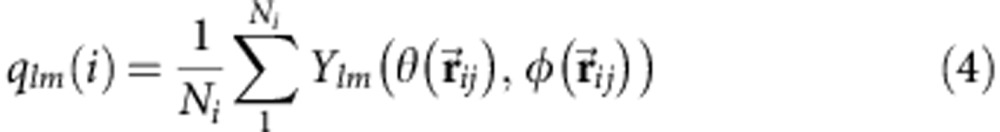


where *Y*_*lm*_ are spherical harmonics and *N*_*i*_ is the number of bonds around atom *i*. The rotational invariants can be defined as:


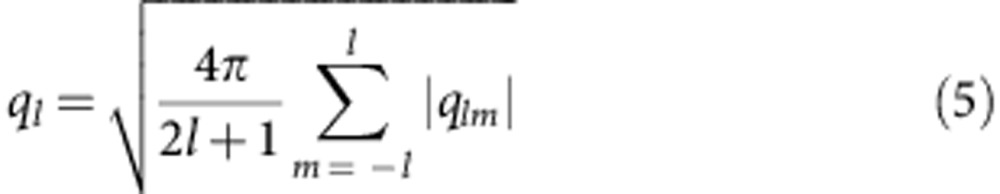


and


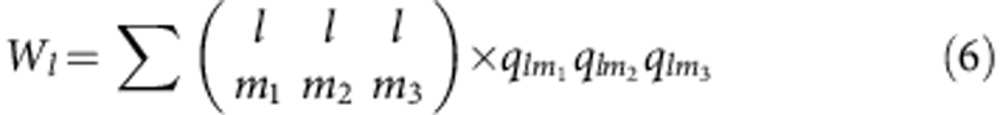


Here the term in bracket in equation (6) is the Wigner 3-*j* symbol. It has been found that *q*_l_ and *W*_*l*_ are the key to a type of cluster shape spectroscopy in liquids and glasses. The normalized parameter 
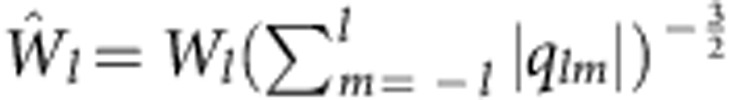
 is a sensitive measure of the different orientational symmetries and often used to evaluate the BOO and differentiate the various local environments^65^. Different atomic clusters may correspond to different values of 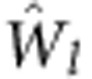. For example, 
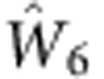
 values of face-centred cubic, hexagonal close-packed and icosahedron clusters are −0.01316, −0.01244 and −0.16975, respectively. Uncertainties in 
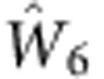
 were estimated from the s.d. of 
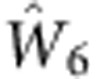
 in two independent MD simulations.

### Voronoi tessellation

Voronoi tessellation divides space into close-packed polyhedral around atoms by constructing bisecting planes along the lines joining the central atom and all its neighbours^66^. The Voronoi index <*n*_3_,*n*_4_,*n*_5_,*n*_6_> is used to characterize the geometry feature of atomic clusters, where *n*_*i*_ (*i*=3,4,5,6) denotes the number of *i*-edged faces of the Voronoi polyhedron. In our analysis, a cutoff distance of 5 Å was chosen so that the Voronoi index distribution was converged^67^.

## Additional information

**How to cite this article:** Xu, W. *et al.* Evidence of liquid–liquid transition in glass-forming La50Al35Ni15 melt above liquidus temperature. *Nat. Commun.* 6:7696 doi: 10.1038/ncomms8696 (2015).

## Supplementary Material

Supplementary Information Supplementary Figures 1-4
