# Supplementary material for: Evidence of liquid–liquid transition in glass-forming La50Al35Ni15 melt above liquidus temperature
Source: Nat Commun. 2015 Jul 13;6:7696. doi: 10.1038/ncomms8696 (PMC4510689; doi:10.1038/ncomms8696)
Supplement: Supplementary Information — Supplementary Figures 1-4 [file ncomms8696-s1.pdf]

Supplementary Figures

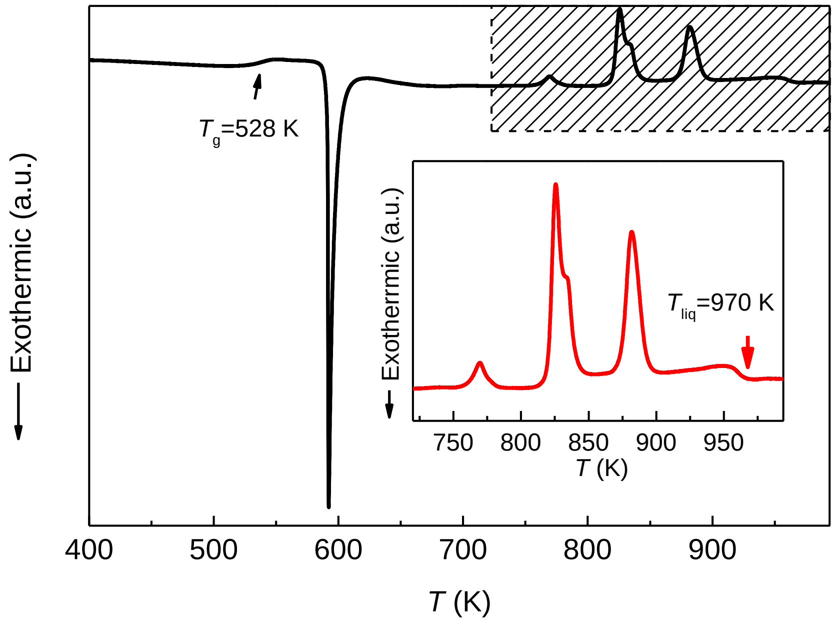

Supplementary Figure 1. DSC trace of as-cast $La_{50}Al_{35}Ni_{15}$ BMG. The DSC curve obtained at a heating rate of 10 K min^{-1}. The onset temperature of glass transition (T_g) is indicated by black arrow. The red line in the inset is a magnified view of the shaded area. Liquidus temperature (T_{liq}) is indicated by red arrow.

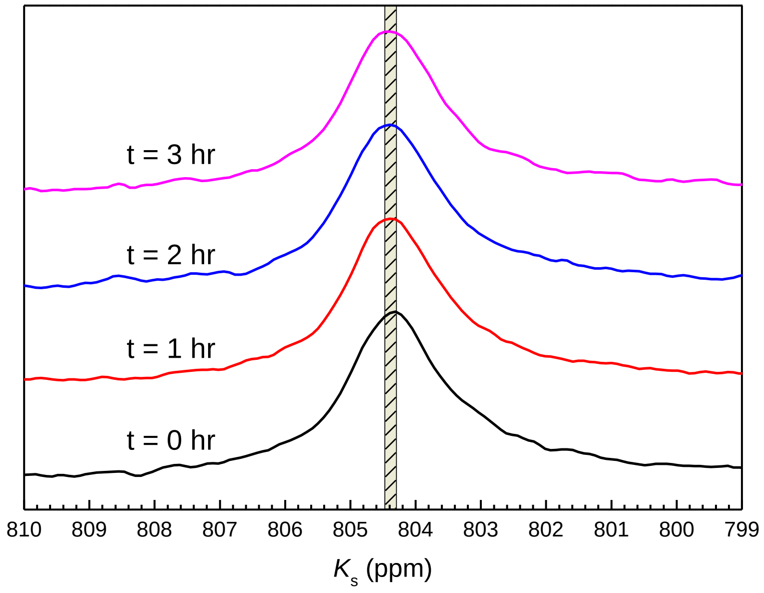

Supplementary Figure 2. Spectrum evolution with time at 1,063 K cooled from 1,143 K. The four NMR spectra were taken at 1,063 K over a time period of 3 hours after cooled from 1,143 K. The same NMR parameters were used as that for continuous measurement shown in Fig. 1b and the inset of Fig. 1d. The spread of the peak position from these separate measurements is shown to be less than 0.2 ppm (shaded area).

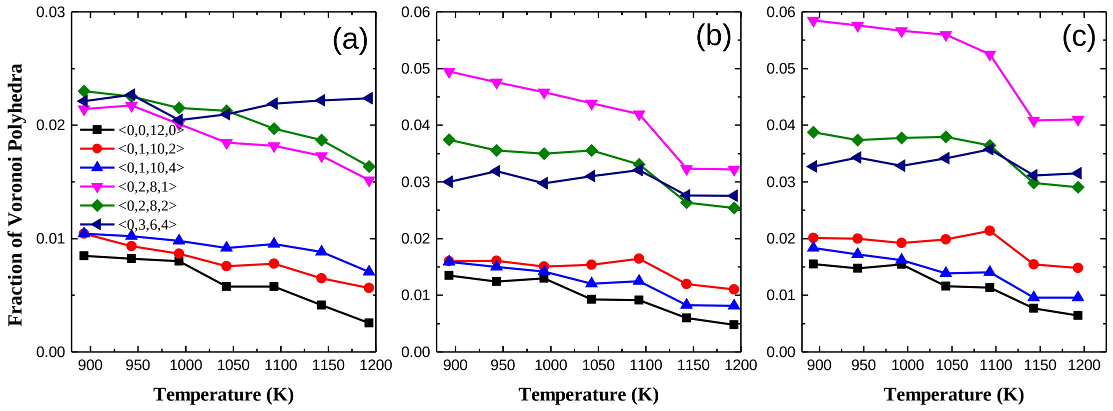

Supplementary Figure 3. The fraction of Voronoi polyhedral without correction (a), with correction of 0.5% (b) and 1% (c), respectively. Analyses removed those surfaces if their areas are less than 0.5% or 1% of the total area of the polyhedron surfaces are added. It is shown that the removal of those surfaces with area less than 0.5% or 1% of the total area of the polyhedron surfaces significantly change the fraction of an individual polyhedra. However, the trend of the population change with temperature does not change much with the small face area correction. Therefore, although the Voronoi analysis for liquids and glasses could lead to ambiguous results, the temperature evolution behavior of the Voronoi polyhedra is consistent once a threshold or criterion is fixed in the analysis.

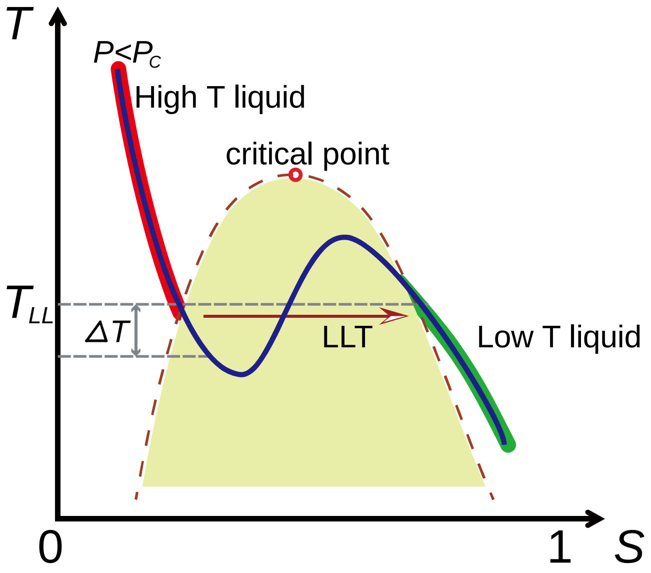

Supplementary Figure 4. The illustration of two-order-parameter model proposed by H. Tanaka. This schematic phase diagram shows the *T* versus *S* curve below the critical pressure ($P < P_c$) calculated from the free energy equation involving two order parameters, namely, density ρ and local bond order parameter *S* characterised by bond orientational order. *S* is a non-conserving order parameter ranging from 0 to 1. A LLT from high temperature liquid (lower *S* value) to low temperature liquid (higher *S* value) can take place during cooling. The undercooling effect with the undercooling degree ΔT could be observed as the curve crosses over the binodal line (dashed line) to the unstable region (lightly green area).
